# Ten-year follow-up results of perioperative chemotherapy with doxorubicin and ifosfamide for high-grade soft-tissue sarcoma of the extremities: Japan Clinical Oncology Group study JCOG0304

**DOI:** 10.1186/s12885-019-6114-2

**Published:** 2019-09-06

**Authors:** Kazuhiro Tanaka, Junki Mizusawa, Norifumi Naka, Akira Kawai, Hirohisa Katagiri, Toru Hiruma, Yoshihiro Matsumoto, Hiroyuki Tsuchiya, Robert Nakayama, Hiroshi Hatano, Makoto Emori, Munenori Watanuki, Yukihiro Yoshida, Takeshi Okamoto, Satoshi Abe, Kunihiro Asanuma, Ryohei Yokoyama, Hiroaki Hiraga, Tsukasa Yonemoto, Takeshi Morii, Keisuke Ae, Akihito Nagano, Hideki Yoshikawa, Haruhiko Fukuda, Toshifumi Ozaki, Yukihide Iwamoto

**Affiliations:** 1 Department of Orthopaedic Surgery, Faculty of Medicine, Oita University, Idaigaoka 1-1, Hasama, Yufu City, Oita 879-5593 Japan; 2 JCOG Data Center, National Cancer Center Hospital, Tokyo, 104-0045 Japan; 3 Musculoskeletal Oncology Service, Osaka International Cancer Institute, Osaka, 541-8567 Japan; 4 Department of Orthopaedic Surgery, National Cancer Center, Tokyo, 104-0045 Japan; 5 Department of Orthopaedic Surgery, Shizuoka Cancer Center, Shizuoka, 411-0934 Japan; 6 Department of Orthopaedic Surgery, Kanagawa Cancer Center, Kanagawa, 241-0815 Japan; 7 Department of Orthopaedic Surgery, Kyushu University, Fukuoka, 812-8582 Japan; 8 Department of Orthopaedic Surgery, Kanazawa University, Ishikawa, 920-8641 Japan; 9 Department of Orthopaedic Surgery, Keio University, Tokyo, 160-0016 Japan; 10 Department of Orthopaedic Surgery, Niigata Cancer Center Hospital, Niigata, 951-8133 Japan; 11 Department of Orthopaedic Surgery, Sapporo Medical University, Sapporo, 060-8556 Japan; 12 Department of Orthopaedic Surgery, Tohoku University, Sendai, 980-8575 Japan; 13 Department of Orthopaedic Surgery, Nihon University, Tokyo, 173-8610 Japan; 14 Department of Orthopaedic Surgery, Kyoto University, Kyoto, 606-8501 Japan; 15 Department of Orthopaedic Surgery, Teikyo University, Tokyo, 173-8606 Japan; 16 Department of Orthopaedic Surgery, Mie University, Mie, 514-8507 Japan; 17 Department of Orthopaedic Surgery, National Kyushu Cancer Center, Fukuoka, 811-1395 Japan; 18 Department of Orthopaedic Surgery, Hokkaido Cancer Center, Sapporo, 003-0804 Japan; 19 Department of Orthopaedic Surgery, Chiba Cancer Center, Chiba, 260-8717 Japan; 20 Department of Orthopaedic Surgery, Kyorin University, Tokyo, 181-8611 Japan; 21 Department of Orthopaedic Surgery, Cancer Institute Hospital, Tokyo, 135-8550 Japan; 22 Department of Orthopaedic Surgery, Gifu University, Gifu, 501-1194 Japan; 23 Department of Orthopaedic Surgery, Osaka University, Osaka, 565-0871 Japan; 24 Department of Orthopaedic Surgery, Okayama University, Okayama, 700-0914 Japan; 25 Kyushu Rosai Hospital, Kitakyushu, 800-0296 Japan

**Keywords:** Soft tissue sarcoma, Extremity, Perioperative chemotherapy, Doxorubicin and ifosfamide, 10-year follow-up, Survival

## Abstract

**Background:**

Soft-tissue sarcomas (STS) are rare malignant tumors those are resistant to chemotherapy. We have previously reported the 3-year follow-up result on the efficacy of perioperative chemotherapy with doxorubicin (DXR) and ifosfamide (IFM) for high-risk STS of the extremities (JCOG0304). In the present study, we analyzed the 10-year follow-up results of JCOG0304.

**Methods:**

Patients with operable, high-risk STS (T2bN0M0, AJCC 6th edition) of the extremities were treated with 3 courses of preoperative and 2 courses of postoperative chemotherapy, which consisted of 60 mg/m^2^ of DXR plus 10 g/m^2^ of IFM over a 3-week interval. The primary study endpoint was progression-free survival (PFS) estimated by Kaplan-Meier methods. Prognostic factors were evaluated by univariable and multivariable Cox proportional hazards model.

**Results:**

A total of 72 patients were enrolled between March 2004 and September 2008, with 70 of these patients being eligible. The median follow-up period was 10.0 years for all eligible patients. Local recurrence and distant metastasis were observed in 5 and 19 patients, respectively. The 10-year PFS was 65.7% (95% CI: 53.4–75.5%) with no PFS events being detected during the last 5 years of follow-up. The 10-year overall survival was 78.1% (95% CI: 66.3–86.2%). Secondary malignancy was detected in 6 patients. The subgroup analysis demonstrated that there was significant difference in survival with regard to primary tumor size.

**Conclusions:**

Only a few long-term results of clinical trials for perioperative chemotherapy treatment of STS have been reported. Our results demonstrate that the 10-year outcome of JCOG0304 for patients with operable, high-risk STS of the extremities was stable and remained favorable during the last 5 years of follow-up.

**Trial registration:**

This trial was registered at the UMIN Clinical Trials Registry as C000000096 on August 30, 2005.

**Electronic supplementary material:**

The online version of this article (10.1186/s12885-019-6114-2) contains supplementary material, which is available to authorized users.

## Background

Soft-tissue sarcomas (STS) are rare types of malignant tumors those account for less than 1% of all malignancies [1]. According to the Soft Tissue Tumor Registry conducted by the Japanese Orthopaedic Association, 1529 patients with STS were registered in 2015 in Japan [2]. The standard treatment for localized, low-grade and/or small STS is surgery. On the other hand, high-grade, large, and deep-seated STS are considered to be high-risk and adjuvant chemotherapy could be a treatment option for this type of STS [3].

We conducted a multicenter phase 2 trial (JCOG0304) of perioperative chemotherapy with doxorubicin (DXR) and ifosfamide (IFM) for treating high-risk STS of the extremities [4]. We have previously reported the perioperative chemotherapy with DXR and IFM was well tolerable and promising in the short-term results of JCOG0304, including the efficacy, safety, and correlation of outcomes with prognostic factors and radiological response [5–7]. In the present study, we analyzed the 10-year follow-up results and report the final results of JCOG0304.

## Methods

From March 2004 to September 2008, a total of 72 patients with high-risk (i.e. high-grade, deep-seated tumors with size > 5 cm), operable STS of the extremities were enrolled in the phase II trial JCOG0304. The study protocol of JCOG0304 was approved by the Protocol Review Committee of the Japan Clinical Oncology Group (JCOG) and was also approved by the Institutional Review Boards of each of the 27 participating institutions. Written informed consent was obtained from each patient before study enrollment.

Details for the eligibility criteria of JCOG0304 have been reported elsewhere [5]. Briefly, the main inclusion criteria were: (1) histologically proven STS of stage III (T2bN0M0), according to the AJCC/UICC 6th edition [8]; (2) histological grade of 2 or 3, according to the French Federation of Cancer Center (FNCLCC) system [9]; (3) resectable tumor in the extremities; (4) age > 20 years, but < 70 years; (5) Eastern Cooperative Oncology Group (ECOG) performance status 0 or 1; and (6) sufficient organ function. The histological diagnosis of biopsy specimens from all patients was reviewed after patient registration by an independent central pathologic committee, which consisted of 3 pathologists who specialized in diagnosing STS.

Preoperative chemotherapy was administrated for 3 courses, followed by surgical resection, and 2 additional courses of postoperative chemotherapy.

Each course of chemotherapy was given every 3 weeks and consisted of DXR (30 mg/m^2^/day on days 1 and 2) and IFM (2 g/m^2^/day on days 1–5). Mesna (1200 mg/m^2^/day) was added after the administration of IFM on days 1–5. Granulocyte-colony stimulating factor was used prophylactically for 7 days if the patient experienced grade 4 neutropenia or leukopenia for more than 5 days after chemotherapy in the previous course.

Surgical treatment was carried out within 5 weeks of the last administration of preoperative chemotherapy. A wide margin should be attempted for the tumor resection; however, a marginal margin was also allowed. If the surgical margin was assessed as insufficient (i.e., margin < 1 cm), a marginal or intralesional margin and local radiation therapy was permitted after the protocol treatment. The decision of administration of radiation was depend on discretionary of the treating physicians, thus the details of radiation therapy were not defined in the protocol of JCOG0304.

The JCOG Data Center conducted data management and the statistical analysis of the present study. Details of the criteria for radiological and pathological response evaluation in JCOG0304 have been reported [5]. Adverse events were evaluated using the National Cancer Institute Common Toxicity Criteria (version 2.0).

### Endpoints and statistical analysis

The primary endpoint of the JCOG0304 study was 2-year progression-free survival (PFS), with secondary endpoints of radiological and pathological response to pre-operative chemotherapy, overall survival (OS), and adverse events. PFS and OS were defined as the time from enrollment to disease progression or death, and to death, respectively. The PFS and OS were estimated by the Kaplan-Meier method. Univariable and multivariable Cox regression analyses were conducted to investigate the prognostic impact of variables on PFS, which included age, sex, histological subtype, tumor differentiation, necrosis, mitosis, histological grade by FNCLCC and Ki-67 system [6], and tumor size. Data assessed by institutional decision was used for univariable analysis only, whereas the data reviewed by the Central Pathological Committee was used for univariable and multivariable analyses. Hazard ratios and *p* values were calculated by Cox regression model. As a sensitivity analysis, multivariable analysis with backward elimination method with alpha of 0.2 was also performed. The difference was considered as significant if the *p* value was < 0.05. All statistical analyses were carried out using SAS software version 9.1 or higher (SAS Institute, Cary, NC, USA).

## Results

### Patients and treatment

**Fig. 1:**
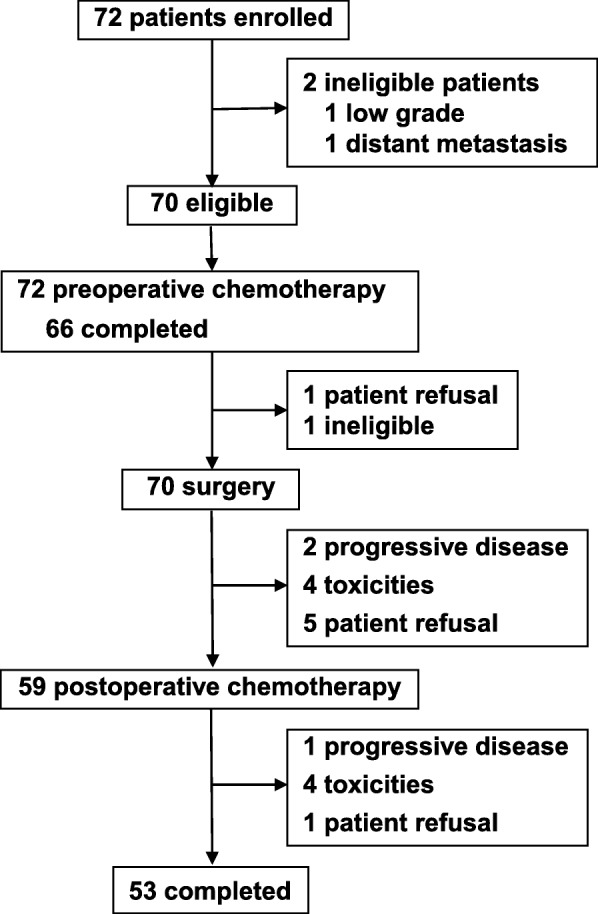
Patient flow diagram

Details of the characteristics of the 72 enrolled and 70 eligible patients have been reported elsewhere [5]. The median follow-up period of all eligible patients beginning from enrollment was 10.0 years (interquartile range, 2.1–11.6 years). The median follow-up duration of the 54 patients without progression or death was 10.4 years (interquartile range, 9.3–to 12.0 years). A diagram of the study flow is shown in Fig. 1. Three courses of preoperative chemotherapy were completed in 66 patients and all 5 courses of the protocol treatment were completed in 53 patients. For local treatment, all eligible patients underwent surgery and 12 patients were treated by adjuvant radiation therapy as a post-protocol treatment.

### Efficacy and safety

**Fig. 2:**
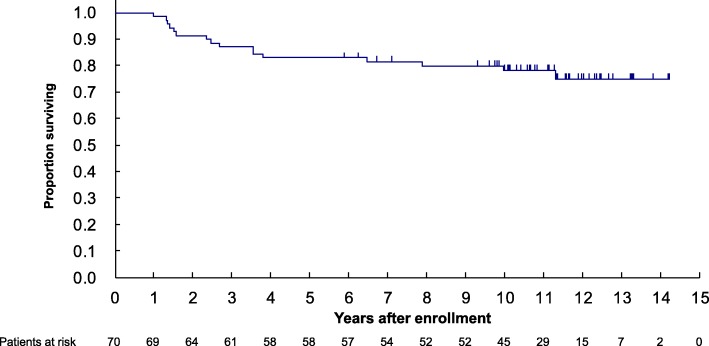
Kaplan-Meier estimate of overall survival

**Fig. 3:**
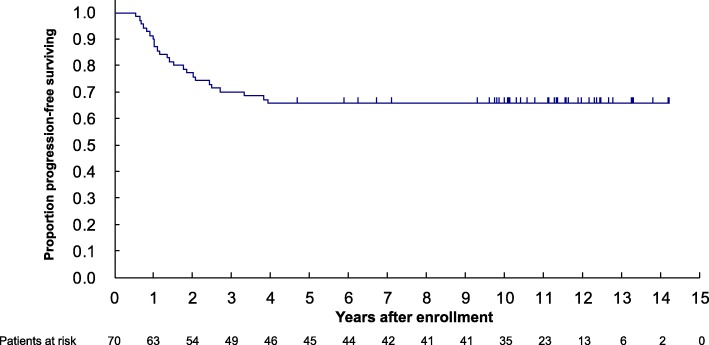
Kaplan-Meier estimate of progression-free survival

When the trial database closed in October 2018, 16 patients had died, with 19 and 5 patients having developed distant and local relapse, respectively. In 12 patients treated by adjuvant radiation therapy, no local recurrence was observed. All 5 patients who had local recurrence were treated by negative margin in surgical resection, and one out of the five patients had died. Compared to the previous reports on JCOG0304 with a median follow-up of 5.1 years, 4 more patients died. In addition, no local or distant relapse were observed within the following 5 years after the data cut-off date for the previous reports. For the 70 eligible cases, the 5- and 10-year OS was 82.9% (95% CI: 71.8–89.9%) and 78.1% (95% CI, 66.3–86.2%), respectively (Fig. 2). Furthermore, the 5- and 10-year PFS were both 65.7% (95% CI: 53.4–75.5%) (Fig. 3).

No treatment related death was observed in the present study. Secondary malignancies were found in 6 patients (breast cancer, chronic myeloid leukemia, prostate cancer, sigmoid colon cancer, lung cancer, gastric malignant lymphoma), however, all of these were considered to be unrelated to the protocol treatment. Regarding late adverse effects, grade 3 ventricular arrhythmia was observed in only 1 patient. Grades 1 and 2 left ventricular dysfunction was observed in 1 patient each. Grade 1 edema, as well as grades 1 and 2 peripheral nerve dysfunction, were found in 5, 4, and 1 patients, respectively.

**Fig. 4:**
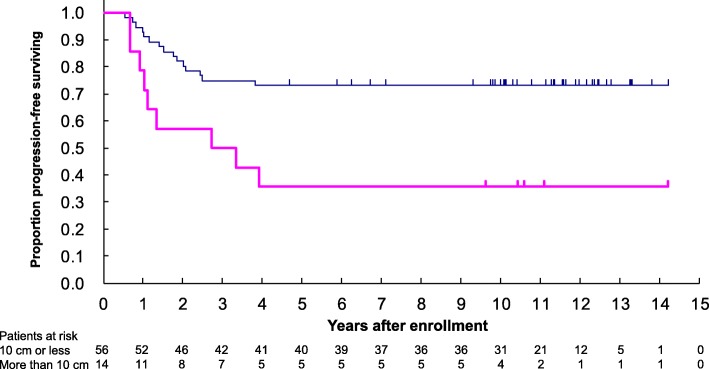
Kaplan-Meier estimate of progression-free survival by primary tumor size

In the univariable analysis, there were no significant differences in PFS regarding sex (male vs. female), age (< 40 vs. ≥40 years), ECOG performance status (0 vs. 1), histological subtype (undifferentiated pleomorphic sarcoma, leiomyosarcoma, synovial sarcoma, or other), histological tumor differentiation (score 3 vs. 2), tumor necrosis (score 1 or 2 vs. 0), and mitosis (score 2 or 3 vs. 1), histological grade evaluated by FNCLCC system (grade 3 vs. 2) and Ki-67 system (grade 3 vs. 2). However, primary tumor size (< 10 cm vs. > 10 cm) significantly influenced on PFS of the patients (Table 1). Furthermore, the multivariable analyses both with all variables and backward elimination demonstrated that there was significant difference in PFS with regard to the tumor size (Fig. 4, Table 2).

**Table 1:** Univariable analysis for progression-free survival

Univariable			
Factors	Category	Univariable analysis	
		HR (95% CI)	*p*-value
Sex	Female (vs. Male)	0.61 (0.27–1.38)	0.23
Age (Years)	≥40 (vs. < 40)	2.04 (0.76–5.47)	0.16
Performance Status	1 (vs. 0)	1.11 (0.46–2.69)	0.81
Histological subtype	Leiomyosarcoma (vs. UPS)	0.59 (0.18–1.98)	0.40
	Synovial sarcoma (vs. UPS)	0.34 (0.10–1.12)	0.08
	Others (vs. UPS)	0.64 (0.24–1.72)	0.38
Tumor differentiation	3 (vs. 2)	0.64 (0.29–1.43)	0.28
Tumor necrosis	1 or 2 (vs. 0)	1.28 (0.56–2.93)	0.56
Tumor mitosis	2 or 3 (vs. 1)	1.14 (0.51–2.57)	0.75
Histological grade (FNCLCC)	Grade 3 (vs. 2)	0.94 (0.42–2.09)	0.87
Histological grade (Ki-67)	Grade 3 (vs. 2)	1.38 (0.61–3.12)	0.43
Tumor size	> 10 cm (vs. < 10 cm)	3.15 (1.37–7.21)	0.0067

*Abbreviations*: *HR* hazard ratio, *CI* confidence interval, *UPS*, undifferentiated pleomorphic sarcoma, *FNCLCC* French Federation of Cancer Center

**Table 2:** Multivariable analysis for progression-free survival

Multivariable			
Factors	Category	Multivariable analysis (including all variables)	
		HR (95% CI)	*p*-value
Sex	Female (vs. Male)	0.70 (0.29–1.72)	0.44
Age (Years)	≥40 (vs. < 40)	1.47 (0.47–4.58)	0.50
Performance Status	1 (vs. 0)	1.41 (0.56–3.59)	0.47
Histological subtype	Leiomyosarcoma (vs. UPS)	0.61 (0.17–2.18)	0.44
	Synovial sarcoma (vs. UPS)	0.48 (0.13–1.80)	0.28
	Others (vs. UPS)	0.65 (0.22–1.87)	0.42
Histological grade (FNCLCC)	Grade 3 (vs. 2)	0.56 (0.17–1.87)	0.34
Histological grade (Ki-67)	Grade 3 (vs. 2)	1.72 (0.51–5.80)	0.39
Tumor size	> 10 cm (vs. < 10 cm)	2.85 (1.18–6.92)	0.0203
Factors	Category	Multivariable analysis (backward elimination with alpha = 20%)	
		HR (95% CI)	*p*-value
Sex	Female (vs. Male)		
Age (Years)	≥40 (vs. < 40)		
Performance Status	1 (vs. 0)		
Histological subtype	Leiomyosarcoma (vs. UPS)		
	Synovial sarcoma (vs. UPS)		
	Others (vs. UPS)		
Histological grade (FNCLCC)	Grade 3 (vs. 2)	0.42 (0.13–1.34)	0.14
Histological grade (Ki-67)	Grade 3 (vs. 2)	2.39 (0.75–7.62)	0.14
Tumor size	> 10 cm (vs. < 10 cm)	3.27 (1.41–7.59)	0.0057

*Abbreviations*: *HR* hazard ratio, *CI* confidence interval, *UPS* undifferentiated pleomorphic sarcoma, *FNCLCC* French Federation of Cancer Center

## Discussion

In the present study, we focused on the long-term results of JCOG0304, as well as the correlations between prognostic factors and outcomes, at a median follow-up of 10 years. Only a few long-term results of clinical trials studying perioperative chemotherapy for STS have been reported due to time and cost issues [10, 11]. As in our previous report, the favorable outcomes of JCOG0304 were maintained during the long-term follow-up of 10 years. We found that perioperative chemotherapy with DXR plus IFM might be useful as a treatment for high-risk STS of the extremities.

In previous clinical trials of adjuvant chemotherapy for STS, it was known that local and distant failure may appear, even as long as 5 years after treatment, and that long-term results of adjuvant chemotherapy tend to lose their initial advantage in survival over surgery alone. For instance, in a randomized controlled trial (RCT) conducted by the Italian Sarcoma Group (ISG) on epirubicin (EPI) and IFM, which compared adjuvant chemotherapy and surgery alone, the 4-year OS of the adjuvant chemotherapy arm was significantly better than that of the surgery alone arm [12]. However, this superiority was lost in subsequent long-term observation and the effectiveness of postoperative adjuvant chemotherapy was considered to be questionable [13].

The ISG conducted another RCT on full-dose EPI and IFM by comparing 5 total courses of pre- and post-operative chemotherapy with 3 courses of preoperative chemotherapy. This trial in which 328 patients with high-risk STS of the extremities and trunk were registered, demonstrated the non-inferiority of the 3-course preoperative chemotherapy for survival [14]. From the long-term observation of the trial, the good 5-year survival results were maintained at the 10-year follow-up [10]. Both the trial of neoadjuvant chemotherapy by ISG and the JCOG0304 study showed long-term stable results. The commonality of both studies is that 3 courses of full-dose anthracycline and IFM have been used prior to surgery. In addition, 91.7% of enrolled patients could complete 3 courses of preoperative chemotherapy. Although both studies lacked a surgery alone arm, the results suggest that preoperative chemotherapy with anthracycline and IFM might be highly effective for treating high-risk STS.

Regarding the usefulness of preoperative chemotherapy for treating high-risk STS, the ISG-STS1001 RCT, which included a 3-course chemotherapy regimen in the neoadjuvant setting, showed interesting results [15]. In this trial, full-dose EPI and IFM as the standard treatment arm and histology-tailored regimens as the experimental arm were compared. The results demonstrated a significantly better 4-year disease-free survival in the standard arm than that of the experimental arm (62 and 38%, respectively, *p* = 0.006), as well as 4-year OS (89 and 64%, respectively, *p* = 0.034). The results in the standard arm were similar to those of the JCOG0304 trial, while those of the histology-tailored chemotherapy arm were similar to those of the surgery alone arm in the study of Frustaci et al. [12]. Moreover, univariable and multivariable analyses in JCOG0304 demonstrated no significant differences in outcomes regarding histological subtype (undifferentiated pleomorphic sarcoma, leiomyosarcoma, synovial sarcoma, or other). So far, the standard treatment with anthracycline and IFM might be favorable for STS of major histological subtypes, at least for those eligible in JCOG0304 and ISG-STS1001.

Since ISG-STS1001 study had a short median follow-up time of 12.3 months, long-term observation is required to confirm these findings. However, compared to ineffective chemotherapy (or surgery alone), the results of the ISG-STS1001 suggested that an improved survival proportion of approximately 20% can be expected for the patients with high-risk STS who receive full-dose anthracycline plus IFM combination chemotherapy administered before surgery [15]. Regarding the efficacy of perioperative chemotherapy for treating STS, a meta-analysis of 18 RCTs that compared surgery alone and adjuvant chemotherapy was performed. The absolute risk reduction for death by adjuvant chemotherapy was found to be 6% (95% CI: 2–11%), while that of DXR plus IFM combination chemotherapy was 11% (95% CI: 3–19%), which demonstrates the usefulness of adding IFM to DXR regimens [16].

After the above-mentioned meta-analysis, the European Organization for Research and Treatment on Cancer (EORTC) conducted a large-scale RCT, EORTC62931, which compared surgery alone and an adjuvant chemotherapy regimen of DXR plus IFM for the treatment of STS. No significant difference between the 2 groups in terms of OS was observed in the trial, which raised doubts regarding the efficacy of adjuvant chemotherapy for treating STS [17]. However, there were several concerns regarding the interpretation of the results of the above trial. In EORTC62931, the IFM dose was low (5 g/m^2^), which was only half that of the ISG trials and JCOG0304. The study enrolled patients with STS of any site or size contained patients (24%) with small tumors (< 5 cm, minimum 0.4 cm). Given these observations, the most useful perioperative chemotherapy regimen currently available is full-dose DXR and IFM combination therapy, and population that would most benefit from this therapy might be high-risk STS of the limb and trunk.

The limitation of the present study is that the JCOG0304 trial was a single-arm phase 2 study and no direct comparison with surgery or postoperative chemotherapy alone was examined. In addition, the number of registered patients was only 72, which can limit the impact of our results.

Taken together with the 10-year results of the present study and the ISG trial, preoperative chemotherapy using full-dose anthracycline and IFM is considered to be useful for treating high-risk STS of the extremities and trunk. It may also be necessary to wait for the final results of the ISG-STS1001 trial. At present, we are conducting a randomized phase 2/3 study of perioperative chemotherapy for the treatment of high-risk STS arising in the extremities and trunk, by comparing DXR plus IFM with gemcitabine plus docetaxel, JCOG1306 [18]. In JCOG1306, we aim to confirm non-inferiority of gemcitabine plus docetaxel to DXR plus IFM since the former regimen is less toxic and has a possibility of combination with other drug including molecular-target therapy in the future therapeutic development.

## Conclusions

The JCOG0304 trial showed a favorable, long-term efficacy of perioperative chemotherapy for treating high-risk STS of the extremities. DXR and IFM might be the most useful perioperative chemotherapy regimen for treating STS. To maximize treatment benefit, full-dose anthracycline plus IFM for patients with high-grade, large and deep-seated STS located in the limbs and trunk may be useful.

## Additional file

Additional file 1: **Table S1.** Names of the Institutional Review Boards of participating institutions. (DOCX 12 kb)

## Data Availability

Individual participant data that underlie the results reported in this Article, after deidentification, will be shared with investigators whose proposed use of the data has been approved by the investigators from the Bone and Soft Tissue Tumor Study Group of the Japan Clinical Oncology Group. Proposals should be directed to the corresponding author.

## Abbreviations

CI: Confidence interval
DXR: doxorubicin
EORTC: European Organisation for Research and Treatment of Cancer
IFM: ifosfamide
JCOG: Japan Clinical Oncology Group
OS: Overall survival
PFS: Progression-free survival
STS: Soft-tissue sarcoma
