# Supplementary material for: Ten-year follow-up results of perioperative chemotherapy with doxorubicin and ifosfamide for high-grade soft-tissue sarcoma of the extremities: Japan Clinical Oncology Group study JCOG0304
Source: BMC Cancer. 2019 Sep 6;19:890. doi: 10.1186/s12885-019-6114-2 (PMC6728960; doi:10.1186/s12885-019-6114-2)
Supplement: Supplementary file 1 — Table S1. Names of the Institutional Review Boards of participating institutions. (DOCX 12 kb) [file 12885_2019_6114_MOESM1_ESM.docx]

**Additional File 1: Table S1. Names of the Institutional Review Boards of participating institutions.**

Sapporo Medical University

Hokkaido Cancer Center

Tohoku University

Chiba Cancer Center

National Cancer Center Hospital

Nihon University

Kyorin University

Keio University

Cancer Institute Hospital

Teikyo University

Kanagawa Cancer Center

Niigata Cancer Center Hospital

Kanazawa University

University of Yamanashi

Gifu University

Shizuoka Cancer Center

Mie University

Kyoto University

Osaka University

Osaka International Cancer Center

Tottori University

Okayama University

Hiroshima Prefectural Hospital

Yamaguchi University

Kanmon Medical Center

Kyushu University

National Kyushu Cancer Center
